# Modeling *In Vivo* Interactions of Engineered Nanoparticles in the Pulmonary Alveolar Lining Fluid

**DOI:** 10.3390/nano5031223

**Published:** 2015-07-22

**Authors:** Dwaipayan Mukherjee, Alexandra Porter, Mary Ryan, Stephan Schwander, Kian Fan Chung, Teresa Tetley, Junfeng Zhang, Panos Georgopoulos

**Affiliations:** 1 Environmental and Occupational Health Sciences Institute (EOHSI), Rutgers University, 170 Frelinghuysen Road, Piscataway, NJ 08854, USA; E-Mail: dp@ccl.rutgers.edu; 2 Department of Environmental and Occupational Medicine, Robert Wood Johnson Medical School, Rutgers University, 170 Frelinghuysen Road, Piscataway, NJ 08854, USA; 3 Department of Chemical and Biochemical Engineering, Rutgers University, 98 Brett Road, Piscataway, NJ 08854, USA; 4 Department of Materials and London Centre of Nanotechnology, Imperial College London, Exhibition Road, London SW7 2AZ, UK; E-Mails: a.porter@imperial.ac.uk (A.P.); m.p.ryan@imperial.ac.uk (M.R.); 5 Department of Environmental and Occupational Health, School of Public Health, Rutgers University, 683 Hoes Lane West, Piscataway, NJ 08854, USA; E-Mail: schwansk@sph.rutgers.edu; 6 National Heart and Lung Institute, Imperial College London, Dovehouse Street, London SW3 6LY, UK; E-Mails: f.chung@imperial.ac.uk (K.F.C.); t.tetley@imperial.ac.uk (T.T.); 7 Nicholas School of the Environment and Duke Global Health Institute, Duke University, 9 Circuit Drive, Durham, NC 27708, USA; E-Mail: junfeng.zhang@duke.edu

**Keywords:** nanoparticles, surfactant, agglomeration, adsorption, lipid vesicles, surfactant proteins, Monte Carlo

## Abstract

Increasing use of engineered nanomaterials (ENMs) in consumer products may result in widespread human inhalation exposures. Due to their high surface area per unit mass, inhaled ENMs interact with multiple components of the pulmonary system, and these interactions affect their ultimate fate in the body. Modeling of ENM transport and clearance *in vivo* has traditionally treated tissues as well-mixed compartments, without consideration of nanoscale interaction and transformation mechanisms. ENM agglomeration, dissolution and transport, along with adsorption of biomolecules, such as surfactant lipids and proteins, cause irreversible changes to ENM morphology and surface properties. The model presented in this article quantifies ENM transformation and transport in the alveolar air to liquid interface and estimates eventual alveolar cell dosimetry. This formulation brings together established concepts from colloidal and surface science, physics, and biochemistry to provide a stochastic framework capable of capturing essential *in vivo* processes in the pulmonary alveolar lining layer. The model has been implemented for *in vitro* solutions with parameters estimated from relevant published *in vitro* measurements and has been extended here to *in vivo* systems simulating human inhalation exposures. Applications are presented for four different ENMs, and relevant kinetic rates are estimated, demonstrating an approach for improving human *in vivo* pulmonary dosimetry.

## 1. Introduction

Engineered nanomaterials (ENMs) are becoming increasingly used in a wide array of consumer products, leading to widespread exposures of human populations [1]. These products include sprays, cosmetics and clothing, greatly enhancing the potential of exposure to ENMs for individuals of all age groups. Based on information available in major ENM databases [1,2], inhalation exposures and dermal exposures represent the two most important exposure routes for nanomaterials that are present in consumer products. Royce *et al.* [3] estimated ENM inhalation exposures from use of representative consumer products across the U.S. population and showed the resulting inhalation exposure to be orders of magnitude higher than the background ambient exposure to the same materials. Inhalation also presents the preeminent route for exposure to airborne particulates, such as pollen, soot, dust and smoke, which are often in the sub-micrometer size range. Such inhalation exposures can lead to a variety of adverse health effects, such as allergic reactions and cardiovascular effects [4].

Inhalation exposure results in a much easier access route for foreign particulate matter to the blood circulation than other routes of exposure, despite the presence of a host of defense mechanisms in the respiratory system. Starting from the mucus layer in the upper airways to the surfactant layer in the alveolar region, along with alveolar epithelial cells and macrophages, inhaled particulate matter interacts with a number of cells and biomolecules following its uptake into the respiratory system. Understanding these interactions, starting with those in the pulmonary alveolar lining, is key to characterizing and quantifying the ultimate biological effects of these exposures. Nanoparticle interactions with the respiratory system involve processes at multiple scales, which require intensive investigation. The pulmonary alveolar lining presents a critical area, where many important interactions take place for nanoparticles. Unlike larger particles, which are preferentially deposited on the walls of the proximal airways, a large proportion of nanoparticles end up in the distal airways and are deposited on the alveolar lining. The interactions of these particles with the alveolar lining fluid lead to a multiscale cascade of effects propagating to the tissue and organ scale [5]. ENMs undergo agglomeration and dissolution in any chemical or biological media, affecting the form, size and surface area of the particles, which ultimately affect the uptake and clearance of the NPs and influence the eventual toxicodynamic effects at the tissue and organism level.

### 1.1. ENM Interactions in Alveolar Lining Fluid

Nanoparticle (NP) transformation has been modeled by multiple researchers using physical theories to assess cellular dosimetry. However, most of these studies have been performed for *in vitro* systems to estimate particle dosimetry to cell cultures. The ISDD (*In vitro* Sedimentation, Diffusion and Dosimetry) model [6] captures NP diffusion and settling for non-interacting particles and their agglomerates. The ADSRM (Agglomeration-Diffusion-Sedimentation-Reaction Model) [7] considers dynamic agglomeration and dissolution, along with diffusion and settling for *in vitro* systems, using a direct simulation Monte Carlo method. The effort described here extends the ADSRM framework to an *in vivo* setting, enabling the assessment of ENM interactions with various fractions of lipids and surfactant proteins, which are present in the respiratory tract as constituents of pulmonary surfactant. One of the most important features of the airway fluid in the alveolar region is the presence of surfactant lipids, which are responsible for reducing surface tension and preventing the collapse of the smallest airways [8]. Alveolar fluid is composed of about 80%–90% lipids, primarily DPPC (dipalmitoylphosphatidylcholine), with the remaining 10% composed of surfactant proteins [5]. The major classes of constituent lipids in pulmonary surfactant are summarized in Table 1. Four surfactant proteins, SP-A, SP-B, SP-C and SP-D, have been identified in the pulmonary alveolar fluid. SP-B and SP-C are known as surface-active proteins and are closely associated with surfactant lipids, assisting in the formation of lipid bilayers at the air-liquid interface [9]. SP-A and SP-D are part of the humoral innate immune system and help in immune response by attaching themselves to foreign particles and identifying them for phagocytosis [5]. These critical components, *i.e.*, phospholipids (PL) and the four surfactant proteins, have been individually considered in this model implementation, as all of them interact with inhaled particulate matter and affect relevant toxicodynamics.

**Table 1:** Properties of alveolar surfactant lipids.

Lipid Species	Percentage composition *	Molecular weight (kDa) **
Phosphatidylcholine (PC)	78	760.076
Phosphatidylethanolamine (PE)	3	471.609
Phosphatidylserine (PS)	5	547.17
Phosphatidylglycerol (PG)	7	787.383
Sphingomyelin (SM)	2	646.505
Cholesterol (CL)	5	400.637

* From *Lung Surfactants*, Notter, 2000 [5]; ** from Avanti Polar Lipids, [10].

### 1.2. Multiscale Toxicodynamics

The work presented here is part of a wider research effort aimed at modeling toxicodynamics of ENMs in mammalian respiratory systems, with the ultimate goal of characterizing human health risks from inhaled ENMs. Mechanisms of toxicodynamic response at the cellular scale are affected by the form, size and surface area of the ENMs [11,12]. It is well known that NP uptake by alveolar cells depends on NP size, surface area and their surface chemistry, particularly adsorption of lipids and proteins on their surface [13]. Consequently, any analysis of the residence time distribution, clearance and biological response of NPs in any tissue system requires a detailed characterization of their form, size and surface reactivity, as these change dynamically with time. The effects of transformation processes were investigated for an *in vitro* culture of alveolar macrophages [14], demonstrating that NP dosimetry to cells is significantly different when relevant transformation processes are considered. Hinderliter *et al.* [6] also showed that the actual amount and form of the NPs reaching the cells over time are markedly different when NP diffusion and settling area are taken into account. Researchers have quantified NP transport, uptake and clearance in the respiratory system [15,16], but have not considered dynamic changes in the form and size of the NPs. Mukherjee *et al.* [17] have also modeled toxicodynamic effects of silver NPs in the mouse pulmonary system, considering effects of surfactant adsorption, secretion, clearance and cellular uptake *in vivo*; however, dynamic transformation of NPs was not considered. The interaction of NPs with lipids and proteins of the lung lining fluid is known to affect their pulmonary toxicodynamics [13,18,19]. Wang *et al.* [20] and Monopoli *et al.* [21] have shown that protein coronas on NPs modify their surface characteristics and are liable to affect their uptake by cells. Wang *et al.* [20] found the protein corona to prevent intracellular NP degradation except inside the lysosome, and so, inclusion of these key mechanisms is essential for any toxicodynamic model for NPs. Blank *et al.* [22] attempted to recreate an alveolar air-liquid interface *in vitro* using human alveolar epithelial cells. However, to date, there have been no published models quantifying ENM transformation *in vivo*. It is almost impossible to realize the exact composition and dynamics of *in vivo* structures and functions *in vitro*. This necessitates detailed *in silico* frameworks that will assemble and integrate relevant *in vitro* and *in vivo* information to produce estimates of *in vivo* toxicodynamic effects. The model developed here uses modules from ADSR Model [7] for diffusion, agglomeration and reaction and introduces separate modules for the adsorption of proteins and lipid vesicles, for its applicability to systems containing lipid and protein molecules, which are known to induce additional transformation mechanisms for the ENMs. The model uses a direct simulation Monte Carlo (DSMC) scheme and estimates various critical kinetic rates pertaining to ENM transformation in the respiratory airway, which can be used to better quantify pulmonary toxicodynamics of ENMs. The approach described here is the first of its kind to include NP to lipid and NP to protein interactions, along with dynamic NP transport and transformation *in vivo*, to predict NP dosimetry in the mammalian respiratory system.

## 2. Methods

Mechanisms of transport and transformation *in vivo* are complicated by the presence of additional biochemical substances and biological defense mechanisms of the tissue concerned. Inhaled particulate matter encounters the following structural and chemical entities in the alveolar region, which act as biological defenses [23]:

- A concentrated layer of pulmonary surfactant, comprising mainly large aggregates of phospholipids (PL) responsible for reducing surface tension at the air-liquid interface;
- A layer of alveolar fluid, which is essentially a mixture of PL (both large and small aggregates), surfactant proteins (SP) and other lipids in an aqueous solution;
- A cellular layer comprising alveolar Type I and Type II cells along with alveolar macrophages with tight junctions between them;
- A basement membrane followed by capillary endothelial cells marking the entrance to the systemic circulation.

The alveolar region is also populated by alveolar lymphocytes (approximately 5%–10% of the bronchoalveolar cells), which play an important role in host defenses within the alveoli [24]. Inhaled ENMs interact differently with each of the entities listed above and, in turn, get transformed by their interactions, which affects their ultimate distribution and toxicity [21]. The reactions of ENMs vary according to their unique material chemistry. Silver ENMs, for which the model was developed and implemented, are generally known to be highly reactive [25,26]. Silver gets readily oxidized in the presence of dissolved oxygen [27] and can also suffer dissolution, as a result of which Ag${}^{\text{+}}$ ions are produced in the medium [25,26]. Oxidative dissolution of Ag ENMs, which was modeled in the ADSRM framework for *in vitro* media [7], has also been considered here. Dissolution is modeled as a function of pH, ionic strength and the concentration of dissolved oxygen in the media. Silver ENMs have also been observed to suffer sulfidation in a variety of environmental media [28,29]. Sulfidation of silver nanowires in human alveolar epithelial cells was observed by Chen *et al.* [30], who showed it to be a potential detoxifying mechanism *in vivo*. Liu *et al.* [29] showed that the kinetics of silver sulfidation are limited by the presence of dissolved oxygen and sulfide groups in the media. The presence of sulfide groups in the alveolar lining fluid being limited, sulfidation is not expected to have a predominant effect on ENM transformation in the lining fluid. However, this is an aspect that needs further investigation, along with the possible formation of other reaction products, such as silver oxides or silver chlorides.

**Figure 1:**
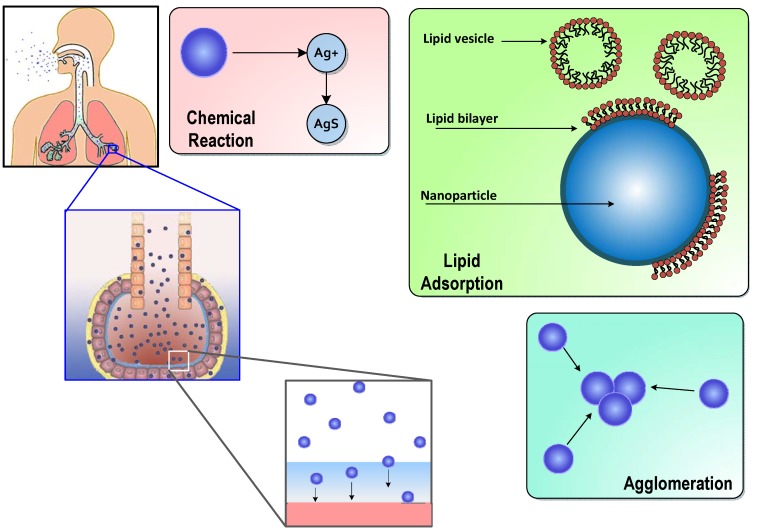
Schematic representation of salient processes and mechanisms of transport, transformation and interaction of inhaled engineered nanomaterials (ENMs) in the pulmonary alveolar lining layer (diagram not to scale).

The model described here employs a direct simulation Monte Carlo (DSMC) method to capture ENM diffusion, settling, agglomeration, reaction and surfactant adsorption *in vivo*. The model is first developed and implemented for *in vitro* lipid solutions, and key parameters are estimated utilizing *in vitro* measurements from the literature. The model considers a slice of the alveolar lining layer and captures the processes taking place after the inhaled ENMs are deposited on the surfactant layer in the alveolar region. The entire set of processes considered in this model is shown schematically in Figure 1.

### 2.1. Surfactant Lipid Adsorption on ENMs

Inhaled particles interact with surfactant lipids from the instant they are deposited on the alveolar surface. By the time the particles arrive at the surface of alveolar cells, they have been modified by the adsorption of lipids and proteins present in the alveolar fluid layer. Bakshi *et al.* [31] and Schleh and Hohlfield [32] have reported the formation of lipid bilayers on NPs immersed in lipid emulsions and have found the thickness of this layer to be approximately 4 nm. Detailed kinetic models for these processes are missing in the literature, because it is difficult to quantify such mechanisms *in vivo*. Adsorption of lipid on to solid surfaces has been traditionally described using Langmuir kinetics [33]. In our previous work (Mukherjee *et al.* [17]), we have described surfactant adsorption on silver ENMs *in vivo* using first-order Langmuir kinetics based on *in vitro* measurements by Kendall *et al.* [13]. However, due to the nanometer size range of ENMs, simple Langmuir kinetics of adsorption is only an approximation [33]. Mornet *et al.* [34] investigated the formation of lipid bilayers on silica NPs and found lipid “patches” on the NPs a few minutes after incubation. They also observed small unilamellar vesicles (SUVs) or liposomes adsorbed on the surface of the NPs, which later underwent deformation and rupture to form lipid bilayers on the NPs. Mornet *et al.* [34] also found that the sizes of these SUVs were in the 10–100 nm range, which makes them comparable in size to ENMs. The interaction of these SUVs with ENMs would be influenced by their mutual surface areas of interaction, their diffusion, and their surface charge distribution, thus making the process inherently heterogeneous, non-uniform and affected by the diffusion of the particles and lipid vesicles.

#### Monte Carlo Simulation of Lipid Vesicle Adsorption

Lipid vesicle diffusion and adsorption on solid supports has been investigated extensively, due to the wide application of “supported lipid bilayers” or SLBs in simulating cell membranes. Zhdanov and co-workers [35,36] have used dynamic MC algorithms to simulate lipid vesicle diffusion, adsorption, deformation and rupture on solid silica wafers immersed in lipid solutions. Their algorithm describes the solid substrate as a square grid and randomly simulates vesicle attachment to grid locations based on the vesicle size and available space on the solid grid. Zhdanov *et al.* also consider inter-vesicle interaction and deformation of vesicles leading to rupture. It is not possible, computationally or otherwise, to implement all of the complexities of the algorithm for multiple ENMs in solution, with each undergoing random motion in the system. Therefore, we have utilized a simpler version of the algorithm employed by Zhdanov *et al.* in which the adsorption of vesicles on to ENMs is based on the sizes of the interacting vesicle and ENM and also on the available area on the ENM. Lipid vesicles are also assumed to deform and rupture immediately after adsorption. The adsorption probability for a random vesicle on to a random ENM is calculated using an exponential equation based on Zhdanov *et al.* [35]:

(1) $$P_{\text{ad}}=exp\left(-\alpha \cdot \frac{d_{\text{ves}}^{\text{2}}}{f_{\text{SA}}^{\text{2}}\cdot d_{\text{ENM}}^{\text{2}}}\right)$$

Here, $d_{\text{ves}}$ is the lipid vesicle diameter, $d_{\text{ENM}}$ is the ENM diameter and $f_{\text{SA}}$ is the fractional exposed (not covered by lipids) surface area of the ENM. The eventual likelihood of adsorption is affected by the diffusion of the vesicles and ENMs and also by the probability of successful adsorption. Zhdanov *et al.* [35] characterized the probability of an ENM and a vesicle colliding due to random diffusion as $P_{\text{diff}}=0.001$ based on a diffusion coefficient of $D=8.7\times 10^{-\text{10}}$ m${}^{\text{2}}$/s [37]. An adsorption attempt is considered successful if a randomly-selected number between 0 and 1 is less than $\frac{P_{\text{ad}}}{P_{\text{ad}}+P_{\text{diff}}}$. The particular steps in the DSMC scheme for lipid adsorption are implemented as follows:

1. A random ENM is selected from the population in the control volume (CV);
2. A random lipid vesicle is selected based on the size distribution of vesicles;
3. $P_{\text{ad}}$ is calculated, such that if the selected ENM has zero exposed surface area, then $P_{\text{ad}}=0$; else $P_{\text{ad}}$ is given by Equation (1);
4. A random number *p* is generated between 0 and 1;
5. Adsorption is considered successful if $p<\frac{P_{\text{ad}}}{P_{\text{ad}}+P_{\text{diff}}}$;
6. If adsorption is successful, the relevant properties of the particular ENM are updated with the changed $f_{\text{SA}}$ and mass;
7. If the maximum number of adsorption steps has not been reached, then the steps are repeated from Step (1).

Gross and Narine [38] found mouse BAL (Bronchoalveolar lavage) fluid to be composed of a 9% “ultra-heavy” large aggregates, a 48% “heavy” fraction with large vesicles and some tubular myelin, and a 43% “light” fraction consisting of small unilamellar vesicles (SUVs). SUVs in alveolar fluid have been reported to vary in size between 20 and 50 nm [5]. Large vesicles (LUVs), which constitute the heavy fraction, are between 50 and 500 nm [5]. A size distribution is constructed for lipid vesicles by fitting a normal distribution within these reported size ranges (limits of the ranges are taken to be the upper and lower 95 percentiles of the distribution). SUVs and LUVs are randomly selected based on their relative fraction in alveolar fluid. Adsorbed lipid vesicles are assumed to rupture after adsorption and to form a lipid bilayer based on the surface area of the initial vesicles. Nordlund *et al.* [39] found only about 1% of the adsorbed vesicles to be intact at the end of adsorption on silica NPs. Various properties of surfactant and its constituent lipids are summarized in Table 2.

**Table 2:** Properties of the airway alveolar lining layer. BALF: bronchoalveolar lavage fluid; PL: phospholipid; DMPC: dimyristoyl phosphocholine.

Property	Value	Reference
BALF density	1.04 g/mL	Shelley, 1975 [40] (study based on male New Zealand rabbits)
Lipid conc.	328 μg/mL	Shelley, 1975 [40] (study based on male New Zealand rabbits)
Protein conc.	212 μg/mL	Shelley, 1975 [40] (study based on male New Zealand rabbits)
BALF vol.	10 mL/kg	Meyer *et al.* [41] (study based on young humans)
PL density	1.108 g/mL	Woodka *et al.* [42] (based on DPPC)
Ionic strength	245 mM	Song *et al.* [43] (study based on 6–8 week CD1 mice)
pH	7.28	Song *et al.* [43] (study based on 6–8 week CD1 mice)
Viscosity	8.79 × 10${}^{-\text{4}}$ kg/(m·s)	Stroumpoulis *et al.* [37] (based on DMPC solution)
Thickness	0.2 μm	Bastacky *et al.* [44] (based on DMPC solution)

### 2.2. Surfactant Protein Adsorption on ENMs

Adsorption of proteins on solid surfaces has been investigated for a number of practical applications. Proteins are part of all biochemical media, and any foreign particle entering the body gets coated with a protein corona, which affects its eventual uptake and/or excretion. Uptake of silver ENMs by pulmonary cells has in fact been shown to be affected by protein corona formation on the particles [21,45,46]. The mechanism of protein adsorption surfaces is known to be composed of the following steps [47]: (1) diffusion of proteins to the surface; (2) actual adsorption on to the surface; and (3) subsequent conformational changes in the protein structure. In the present model, the first two steps are explicitly considered. Modeling conformational changes in protein structure would require a detailed proteomics model, which is beyond the scope of this work. Pino *et al.* [46] have studied the kinetics of protein adsorption and desorption on the surface of NPs. Zhdanov and Kasemo [48] have described a Monte Carlo (MC) model for protein adsorption on solid surfaces, which has been extended here for ENMs in solution. Two separate cases are considered: protein adsorption (1) under a diffusion-controlled regime and (2) under an adsorption-controlled regime. The model algorithm selects the case based on the slower or limiting process based on the conditions of the system at any given time. Under the adsorption-limited regime, the adsorption probability is given by:

(2) $$P_{\text{Ad}}=2R^{\text{2}}C_{\text{o}}k_{\text{a}}$$

where *R* is the radius of the particular protein headgroup, $C_{\text{o}}$ is the concentration of the protein in solution and $k_{\text{a}}$ is the adsorption rate for the particular protein. The time step associated with the adsorption process is given by $\Delta t=\frac{1}{P_{\text{Ad}}}$. Under the diffusion-limited regime, the adsorption probability is given by:

(3) $$P_{\text{Ad}}=2R^{\text{2}}C_{\text{o}}\sqrt{\pi Dt}$$

where *D* is the diffusivity of the protein in the solution and *t* is the system time. In this case, the time step associated with the process is given by $\Delta t=\frac{\sqrt{\pi t/D}}{C_{\text{o}}A}$, where *A* is the protein headgroup area. Adsorption of proteins is however not irreversible and is actually a net result of adsorption and desorption [36]. Furthermore, adsorption of proteins cannot be independent of adsorption of other chemical species, as the charged headgroups of proteins are liable to interact with charges already present on the ENM surface. Lipid bilayers are known to interact with proteins through electrostatic interaction and, thus, affect the adsorption and clustering of protein molecules [49]. Electrostatic interaction and the number of charged-groups in contact would affect adsorption and the subsequent desorption of proteins from surfaces [36]. Zhdanov and Kasemo [36] modeled protein adsorption and desorption on solid surfaces in the presence of lipid bilayers and measured adsorption rates, $k_{\text{a}}$, and desorption rates, $k_{\text{d}}$, for various values of surface coverage by lipid bilayers. Adsorption and desorption of surfactant proteins have been considered in this model as functions of ENM surface coverage. A power-law model was fitted to the measurements by Zhdanov and Kasemo [36] to estimate $k_{\text{a}}$ and $k_{\text{d}}$ as:

(4) $$\begin{array}{cc} k_{\text{a}} & =k_{\text{a}}^{\text{0}}\cdot \left(1+{\text{β}}_{\text{a}}\cdot {\text{θ}}^{na}\right) \end{array}$$

(5) $$\begin{array}{cc} k_{\text{d}} & =k_{\text{d}}^{\text{0}}\cdot \left(1+{\text{β}}_{\text{d}}\cdot {\text{θ}}^{nd}\right) \end{array}$$

where $k_{\text{a}}$, $k_{\text{d}}$ are the adsorption and desorption rates for the proteins on to ENMs, θ is the fractional surface coverage of ENMs by lipids and β and *n* are fitted parameters for adsorption and desorption. Details of the estimation of these parameters for different SPs are included in Supplementary Materials. A net rate defined as $k_{\text{a}}^{\prime }=k_{\text{a}}/k_{\text{d}}$ was used in Equation (2) to simulate adsorption, so that the effects of both adsorption and desorption could be simultaneously taken into account in the DSMC framework. Pulmonary surfactant is composed of four types of proteins: SP-A, -B, -C and -D. The first three types of proteins have been investigated considerably, but relatively little information is available for SP-D. SP-A is the most abundant protein in pulmonary surfactant, accounting for 5% by weight of surfactant, followed by SP-B and SP-C, which together account for 1.5% by weight [5]. Detailed properties of the protein molecules have been summarized in Table 3.

**Table 3:** Properties of surfactant proteins.

Protein	MW (kDa) *	Length (nm) *	Dia. (nm) *	Density (g/cm${}^{\text{3}}$) ${}^{\text{+}}$	Conc. (μg/mL) **	D (10${}^{\text{9}}$ m${}^{\text{2}}$/s)${}^{\text{++}}$
SP-A	32	20	20	1.41	132.5	0.093
SP-B	9	7.9	3	1.48	19.93	0.5474
SP-C	3.8	3.4	2	1.5	19.93	0.796
SP-D	42	92	92	1.4	39.64	0.0206

* From *Lung Surfactants*, Notter, 2000 [5]; ${}^{+}$ from Fischer *et al.* [50]; ** based on the percentage composition from Notter [5] and total protein conc. in pulmonary alveolar fluid from Shelley, 1975 [40]; ${}^{++}$ diffusion coefficient estimated using the Svedberg equation [51] (details in Supplementary Materials).

### 2.3. ENM Transport in Alveolar Lining Fluid

NP diffusion and settling have been modeled by Hinderliter *et al.* [6] (ISDD Model) and also recently for silver ENMs by Mukherjee *et al.* [7] (ADSR Model) for *in vitro* media. As shown in both articles, gravitational settling has a major effect on ENM settling and eventual dosimetry for *in vitro* cell cultures. However, it has been shown that, for nanoscale particles *in vivo*, the forces of surface tension exerted by the surface-active alveolar lining layer are 2–3 orders of magnitude greater than gravitational forces [52]. Accordingly, gravitational forces have been replaced by surface tension for the *in vivo* implementation of the model. For the *in vitro* implementations in this work, gravitational settling has been modeled using Stokes’ law in a manner similar to Hinderliter *et al.* [6]. Surface forces acting on small particles in the alveolar lining were mathematically analyzed by Schurch *et al.* [52]. The net force that causes the immersion of the particles into the alveolar fluid is calculated as [52]:

(6) $$F_{\text{s}}=2\pi R\text{γ}\cdot sin\left(\text{ϕ}\right)\cdot sin\left(\text{θ}+\text{ϕ}\right)$$

where *R* is the particle radius, γ is the surface tension at the alveolar air-liquid interface, θ is the contact angle at the particle surface and ϕ is the angle formed between the contact point and the axis of the particle. The surface force $F_{\text{s}}$ can be equated to the drag force in the medium using Stokes’ law to obtain:

(7) $$v=\frac{\text{γ}}{3\text{μ}}\cdot sin\left(\text{ϕ}\right)\cdot sin\left(\text{θ}+\text{ϕ}\right)$$

Schurch *et al.* [52] found that for low values of surface tension (*γ* = 30 dyn/cm), such as those experienced at the alveolar lining, the contact angle θ would have a value of 20${}^{\circ }$ and that the value of ϕ would be about 160${}^{\circ }$.

### 2.4. ENM Agglomeration

ENM agglomeration has been modeled in a fashion similar to that in Mukherjee *et al.* [7] by considering modified versions of Smoluchowski's equations. However, lipid adsorption on the ENMs causes surface modification, which has been found to dissuade agglomeration in silver ENMs [26]. This has been taken into account by using the values of the zeta potential estimated by Leo *et al.* [26] for DPPC-adsorbed silver ENMs. The zeta potential has been considered as a weighted function of surface coverage by phospholipids as:

(8) $$\zeta =\zeta_{\text{L}}\cdot \text{θ}+\zeta_{\text{o}}\left(1-\text{θ}\right)$$

where $\zeta_{\text{o}}$ is the zeta potential of the ENM without lipid adsorption, $\zeta_{\text{L}}$ is the reduced zeta potential due to lipid adsorption and θ is the fractional surface coverage of the ENM by lipids. The reduced zeta potential $\zeta_{\text{L}}$ due to lipid adsorption is estimated from the data of Leo *et al.* [26], where zeta potentials of silver NPs were measured with and without the addition of DPPC. ENM agglomeration is also known to be affected by steric effects due to the presence of coating molecules. Leo *et al.* [26] reported that despite a reduction in zeta potential, the addition of DPPC maintained the dispersed state of NPs and did not promote agglomeration due to steric effects of the coating molecules. There was no such effect in the results of Nordlund *et al.* [39], where the addition of lipids led to increased agglomeration. This phenomenon was also observed by Kendall *et al.* [19] for particulate matter. This has been reconciled in this model by introducing steric effects for coating molecules, which would produce additional repulsive interactive forces. Damodaran [53] modeled repulsive interactions due to steric effects in proteins by considering a repulsive energy due to steric effects that can be represented as:

(9) $$E_{\text{st}}=\left(kTn_{\text{m}}L/s\right)\cdot \left[{\left(2L/d\right)}^{\text{2.25}}-{\left(d/2L\right)}^{\text{0.75}}\right]$$

where *k* is the Boltzmann constant, *T* is the absolute temperature, $n_{\text{m}}$ is the number of coating molecules per unit surface area of the ENM, *L* is the chain length of the coating molecule, $s=\sqrt{1/n_{\text{m}}}$ is the mean distance between coating molecules and *d* is the mean distance between interacting ENMs. The steric repulsive energy (details in Supplementary Materials) is included in the calculation of repulsive interactions as part of Smoluchowski's equations, which consider both attractive van der Waals’ forces and repulsive electrostatic forces of interaction.

## 3. Results and Discussion

The DSMC algorithm is implemented in a constant number mode. A population of 100 ENMs is selected, and a control volume (CV) is defined, which contains these ENMs based on the concentration of the system. At all times during the simulation, a number of internal and external coordinates of the system of ENMs is recorded. The internal coordinates of ENMs refer to various dynamic properties of the particles, such as position, diameter, surface coverage by lipid, adsorbed proteins and agglomeration state. External coordinates refer to parameters of the medium, such as lipid and protein concentration, ENM number concentration, temperature, density, viscosity, *etc*. Results of the ADSR Model implementation for ENM transport and transformation are presented next, in two parts. The first part concerns model implementation *in vitro* and estimation of key parameters pertaining to ENM agglomeration and lipid adsorption. The model has been implemented for *in vitro* lipid solutions, and simulation results are compared with measurements reported by Leo *et al.* [26] for silver NPs and Nordlund *et al.* [39] for silica NPs. In the second part, the model has been implemented for the human alveolar lining layer, and predictions of the model are presented for *in vivo* ENM transformation. Various critical kinetic rates are also estimated based on the *in vivo* results.

### 3.1. *In Vitro* Implementations

The ADSR Model described in the present article was implemented for an *in vitro* system based on the works by Leo *et al.* [26] and Nordlund *et al.* [39]. Some features of the model implementation were exclusively applied for the *in vitro* cases as follows:

- Lipid concentration is considered to vary dynamically from the initial values in solution. Vesicle diffusivity was appropriately modified. For *in vivo* systems, the levels of lipid in alveolar lining are maintained relatively constant by alveolar cells via constant secretion and removal.
- DPPC vesicle size distribution and concentration have been considered based on the commercially available formulations used in the relevant studies, rather than on the properties of actual biological surfactant.
- Gravitational settling has been considered, rather than surface-active transport, unlike the situation in the actual alveolar lining, where surface tension-aided transport far outweighs gravitational transport (discussed in the Methods).

**Figure 2:**
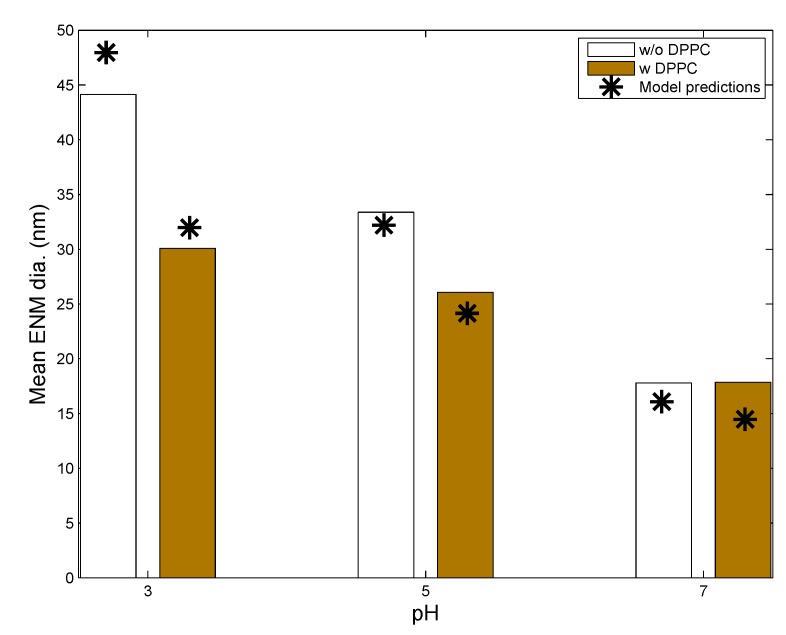
Comparison of model predictions of silver ENM mean diameter (shown as stars) with *in vitro* measurements (shown by bars) from Leo *et al.* [26] for citrate-coated silver ENMs (L20; initial mean diameter = 20 nm; other ENM properties in Table 5) incubated with and without dipalmitoylphosphatidylcholine (DPPC) for 7 days.

**Figure 3:**
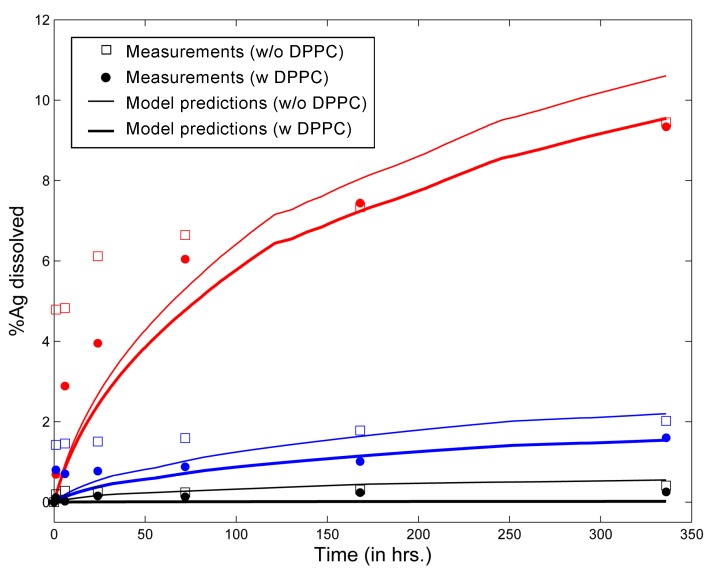
Comparison of model predictions of percent Ag dissolved in solution with *in vitro* measurements from Leo *et al.* [26] for citrate-coated silver ENMs (L20; initial mean diameter = 20 nm; other ENM properties in Table 5) incubated with and without DPPC for up to 14 days (results for pH 3 in red, for pH 5 in blue and for pH 7 in black).

Leo *et al.* [26] used citrate-coated silver NPs (AgNPs) in solution (with and without DPPC) at varying values of pH and followed the change in particle size and silver dissolution over a span of seven days. Values of various ENM and media properties were appropriately selected to mimic the *in vitro* system used by Leo *et al.*, and these are summarized in Table 4. Figure 2 shows comparisons between model predictions and *in vitro* measurements from Leo *et al.* [26] for the mean diameter of AgNPs in solution after seven days of incubation in media of different pH, with and without the addition of DPPC. Mean diameter has been calculated considering the entire particle population from *in vitro* measurements reported by Leo *et al.* and taking the number-weighted mean of the particle diameters. The model captures the phenomenon of increased agglomeration due to lower values of pH reasonably well. The measurements of Leo *et al.* also demonstrate decreased agglomeration due to DPPC despite the lowering of surface zeta potentials due to lipid adsorption. This has been attributed to the influence of steric factors. Accordingly, a steric factor has been included in the estimation of repulsive interaction energies for coated ENMs through Equation (9). Appropriate modulation of values of the adsorption probability *α* in Equation (1) and the steric factor, $\eta_{\text{S}}$ in Equation (9), allows the model results to match the *in vitro* measurements. The final values of the estimated parameters are summarized in Table 4. Figure 3 compares model predictions and *in vitro* measurements for dissolution of AgNPs in media with and without DPPC. The model shows reduced dissolution when DPPC is added due to increased surface coverage of the ENMs by DPPC molecules, which reduces the rate of silver oxidation and dissolution from the AgNP surface. Furthermore, the inhibitory effect of DPPC on Ag dissolution increases with time, as AgNP surface coverage increases. It is also seen that the effect of lipid adsorption is much less than the effect of pH, most probably due to gravitational settling. AgNPs with large amounts of adsorbed DPPC have a greater tendency to settle and are thus removed from the solution.

**Figure 4:**
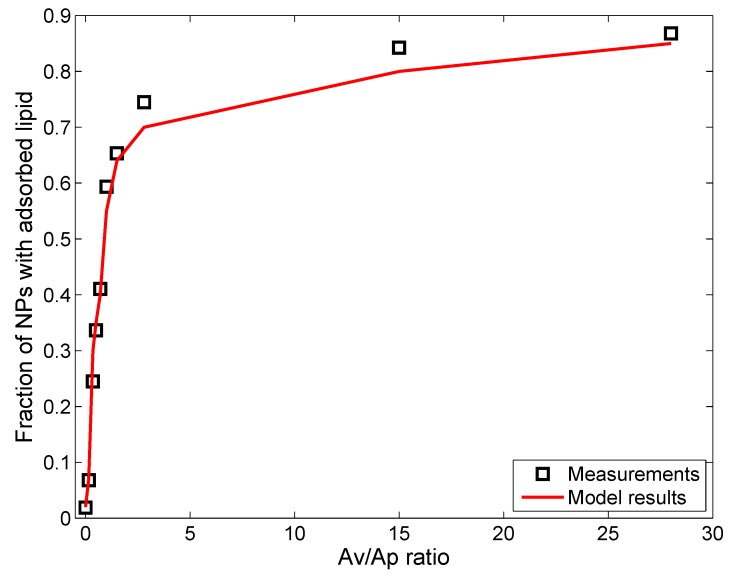
Comparison of model predictions (red line) of fraction of NPs (N600; initial mean diameter = 600 nm; other ENM properties in Table 5) with adsorbed lipids after 1 h of incubation, with *in vitro* measurements (black squares) from Nordlund *et al.* [39] for different values of surface area ratios of lipid vesicle to particle.

**Figure 5:**
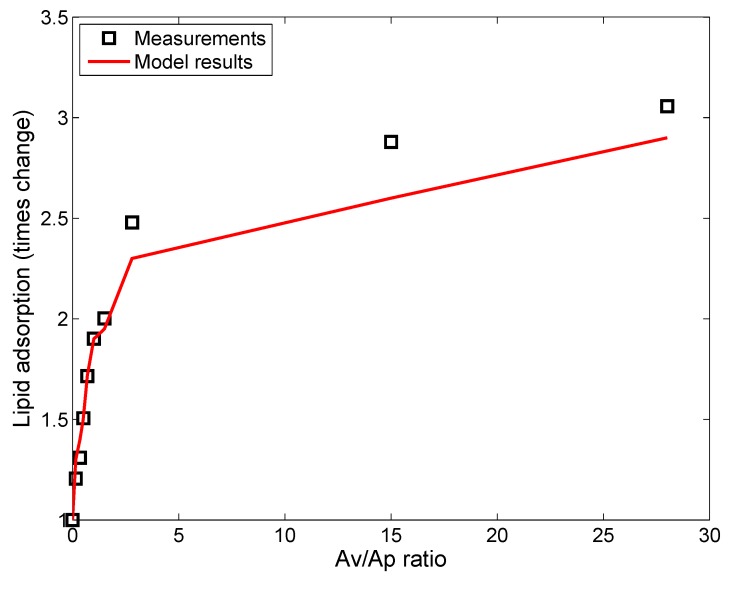
Comparison of model predictions (red line) of the time change in the amount of adsorbed lipids after 1 h of incubation, with *in vitro* measurements (black squares) from Nordlund *et al.* [39] for different values of surface area ratios of lipid vesicle to NP (N600; initial mean diameter = 600 nm; other ENM properties in Table 5).

**Figure 6:**
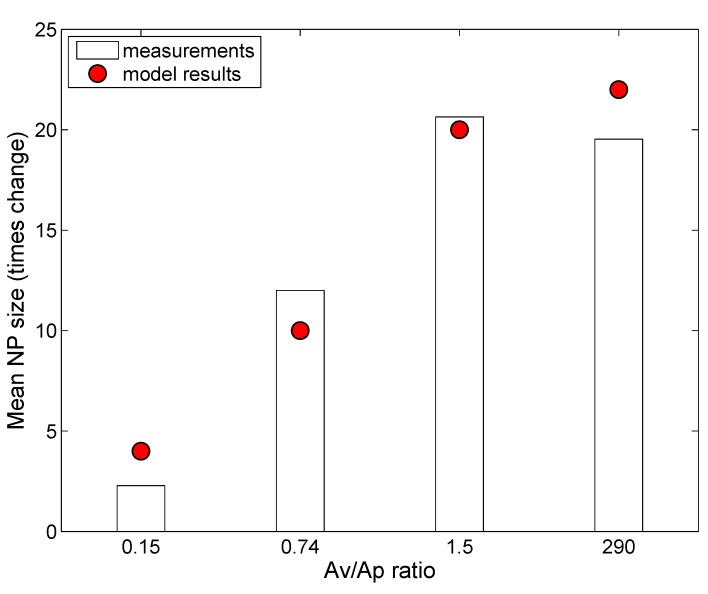
Comparison of model predictions (shown as stars) of the average size of silica NPs (N600; initial mean diameter = 600 nm; other ENM properties in Table 5) after one hour of incubation with *in vitro* measurements (shown as bars) from Nordlund *et al.* [39] for different values of surface area ratios of lipid vesicle to particle.

Nordlund *et al.* [39] used silica NPs (SiNPs) (without coating) to study lipid adsorption from a solution of DOPC (1,2-dioleoyl-*sn*-glycero-3-phosphocholine). DOPC has a similar zwitterionic polar headgroup as DPPC, with oppositely-charged amine and phosphorus groups next to each other [10]. Interactions between DOPC molecules and negatively-charged SiNPs are expected to be similar to those between DPPC and citrate-stabilized AgNPs. However, as reported by Leo *et al.* [26], the presence of citrate groups might add to the steric effects, which are absent in the case of SiNPs. In fact, Nordlund *et al.* [39] reported that the SiNPs have an increased tendency to agglomerate in the presence of DOPC, which suggests lesser steric effects than for NPs with coating. The ADSRM model was implemented for the *in vitro* system corresponding to the study with SiNPs and with different values of lipid-to-NP ratios as performed by Nordlund *et al.* [39]. However, in that study, the SiNPs were incubated with DOPC vesicles for 1 h, accompanied by stirring. This would prevent settling of particles, and, accordingly, settling was not considered here. Appropriate parameter values required for this implementation are summarized in Table 4. In the study by Nordlund *et al.* [39], DOPC was mixed with fluorescein, and the associated fluorescence on the SiNPs due to adsorbed DOPC was measured by flow cytometric analysis. Figure 4 compares model predictions of fraction of particles with adsorbed DOPC and *in vitro* measurements from Nordlund *et al.* [39] for ten values of $A_{\text{v}}/A_{\text{p}}$, which represent the initial surface area ratios of vesicle to NP. Figure 5 compares the predicted average amount of DOPC adsorbed on SiNPs in solution with *in vitro* measurements of average fluorescence from Nordlund *et al.* [39]. The results have been normalized by the value of mean fluorescence with no DOPC added to correct for fluorescence produced by SiNPs themselves. This model implementation has been carried out along with the appropriate selection of values for adsorption probability α in Equation (1) and the steric factor, $\eta_{\text{S}}$ in Equation (9). $\eta_{\text{S}}$ has a lower value than that in the earlier implementation due to the absence of coating on the SiNPs. Figure 6 compares the model-predicted average diameter of SiNPs in solution with measurements of fluorescence with increasing vesicle-to-particle surface area ratios. Average diameter has been calculated by considering the entire particle population from *in vitro* measurements reported by Nordlund *et al.* and taking the number-weighted mean of the particle diameters.

**Table 4:** Parameters used for model implementations.

Parameters	Leo *et al.*, 2013 [26]	Nordlund *et al.*, 2009 [39]	*In Vivo* Implementation
System	Eppendorf tube	NR	Alveolar lining
System volume	4 mL	NR	10 mL/kg BW [41]
ENM type	Ag NP	Silica NP	Ag NP
ENM coating type	Citrate (C)	None	Citrate (C), PVP
Coating molecular weight (mol. wt.)	258	None	258 (C), 10,000 (PVP)
Initial ENM diameter (nm)	20	600	20, 110
ENM density (g/mL)	10.49	2	10.49
ENM concentration (mg/L)	25	3000	NA *
Ionic strength (mM)	0.1	100	245 [43]
pH	3, 5, 7	7.4	7.28 [43]
Initial ENM zeta potential (mV)	−18.2, −22.5, −32.5	−25 [54]	−32.5
Dissolved O${}_{\text{2}}$ (mg/L) **	8.96	8.96	8.96
Temperature	37 ${}^{\circ }$C	22 ${}^{\circ }$C	37 ${}^{\circ }$C
Adsorption probability, *α*	0.004	0.001	0.004
Steric factor, ${\text{η}}_{\text{S}}$	2.35	1	2.35 (C), 10.6 (PVP)

* *In vivo* dosage was simulated as inhaled aerosolized ENM present in consumer products [3]; ** saturated O${}_{\text{2}}$ conc. for solutions exposed to atmospheric pressure [25]; values, unless otherwise referenced, are from the original studies; NR = not reported.

**Table 5:** Properties of silver ENMs used in the model implementations.

NP	Coating	Core Material	Density (g/cm${}^{\text{3}}$)	Mol. Wt.	Coating Mol. Wt.	Zeta Potential (mV)	Ref.
L20	Citrate	Ag	10.49	108	258	−39.2	[26]
N600	None	SiO${}_{\text{2}}$	10.49	108	258	−39.2	[39]
C20	Citrate	Au	10.87	115.3	258	−44.3	[7,55]
P20	PVP	Au	10.87	115.3	10,000	−38.2	[7,55]
C110	Citrate	Au	10.49	108.04	258	−45.2	[7,55]
P110	PVP	Au	10.49	108.04	40,000	−31.6	[7,55]

### 3.2. *In Vivo* Implementation

The ADSR Model developed in the present article was also implemented for the human alveolar lining layer, simulating ENM dosimetry after inhalation exposure. This formulation specifically helps us estimate key kinetic parameters of ENM transformation and transport in the alveolar lining, which can inform toxicodynamic models relevant to the human pulmonary system. The following modifications were incorporated into the model to reflect conditions relevant to the *in vivo* system:

- The ENM dose to the alveolar region is based on an inhalation dose for an adult human.
- Airway dosimetry of ENMs based on alveolar deposition is estimated by the software MPPD [56].
- Gravitational settling is replaced by surface-tension-assisted transport of particles towards the cellular layer.
- The concentration of lipids and proteins is considered constant due to homeostasis maintained by constant secretion from alveolar cells.

Various parameters of the airway and the alveolar lining layer are summarized in Table 2. ENM dose is considered to be delivered by a single breath by an adult human, breathing in an ENM cloud with a reference concentration of 0.14 mg/m${}^{\text{3}}$. The reference concentration is obtained from Royce *et al.* [3] and is based on the maximum concentration in the immediate breathing zone after spraying of a bathroom cleaner containing silver ENMs in an indoor residential microenvironment. The mass concentration is converted to the number concentration based on the size distribution of the ENMs used, and the ENMs are all considered to be monomers initially. Four types of silver ENMs are used in this implementation to test the effect of ENM and coating type, specifically 20- and 110-nm silver ENMs with citrate and PVP (polyvinyl pyrrolidone) coatings. Wang *et al.* [55] used these four types of NPs to test their dosimetry in cell culture plates. The ENMs are referred to as C20 , C110, P20 and P110, respectively, and their properties are summarized in Table 5. Human airway dosimetry modeling is performed using the MPPD software package [56], which considers age-dependent changes in airway morphology to calculate particle deposition based on particle size and density in different regions of the lung. The entire population of ENMs reaching the alveolar region is divided by the known air-exchange area of alveoli (70 m${}^{\text{2}}$ [32]) in an adult human lung. Various parameters of the mammalian alveolar lining layer are summarized in Table 2. The ADSR Model is implemented through a constant number DSMC algorithm consisting of the following steps:

1. A constant number (*N* = 100) of ENMs is selected, and the associated alveolar area is calculated.
2. The volume of the starting control volume (CV) is calculated based on a uniform average thickness of the alveolar lining layer.
3. The ENMs are initially all considered to be deposited at the top of the CV.
4. ENMs are considered to be monomers, as there is negligible chance for agglomeration in the airway before coming in contact with a surface.
5. Diameters and all other internal coordinates of the ENMs in the CV are estimated.
6. Various modules of the model—agglomeration, transport, adsorption, desorption and reaction—are successively implemented as described in detail in the Methods Section.
7. Time is advanced by Δt based on the slowest step among all processes.
8. New CV is established based on the constant number *N*.
9. ENM and media property values are saved, and the steps are run again from Step 5 as described above.

The model simulation stops when the concentration of ENMs in the alveolar lining layer drops to 1% or less of its initial concentration. Since the transport of ENMs through the alveolar lining layer is a relatively fast process, compared to the earlier implementations for *in vitro* media, the model involves a relatively small number of steps to complete. The bottom of the alveolar lining and the location of the alveolar cellular surface is considered the bottom of the CV and where the ENMs settle. The current model implementation predicts the state, number and condition (adsorbed surfactant lipids and proteins) of ENMs present at the bottom of the lining layer after inhalation, which would ultimately affect their uptake by the alveolar cells and macrophages.

#### 3.2.1. Estimation of Kinetic Parameters

**Figure 7:**
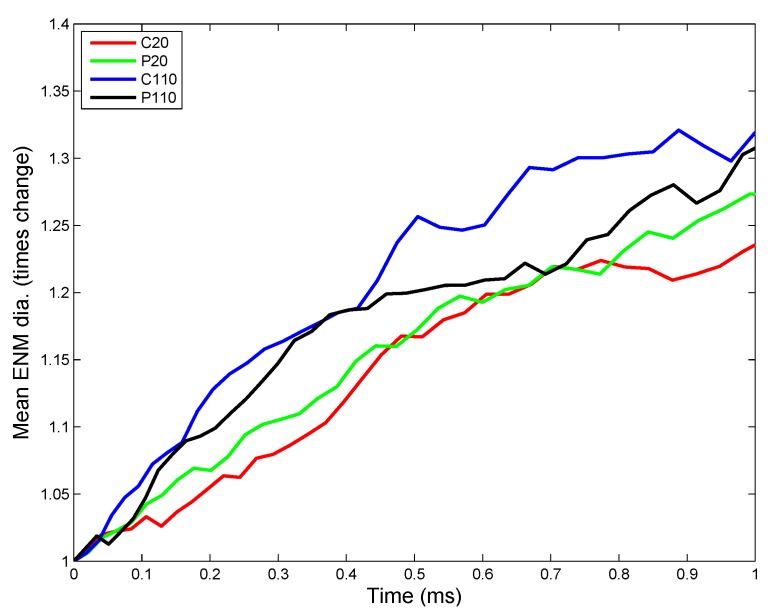
ADSRM simulation results of the change in mean ENM diameter (times initial mean diameter) for four different types of silver ENMs of diameters of 20 and 110 nm (other ENM properties in Table 5).

**Figure 8:**
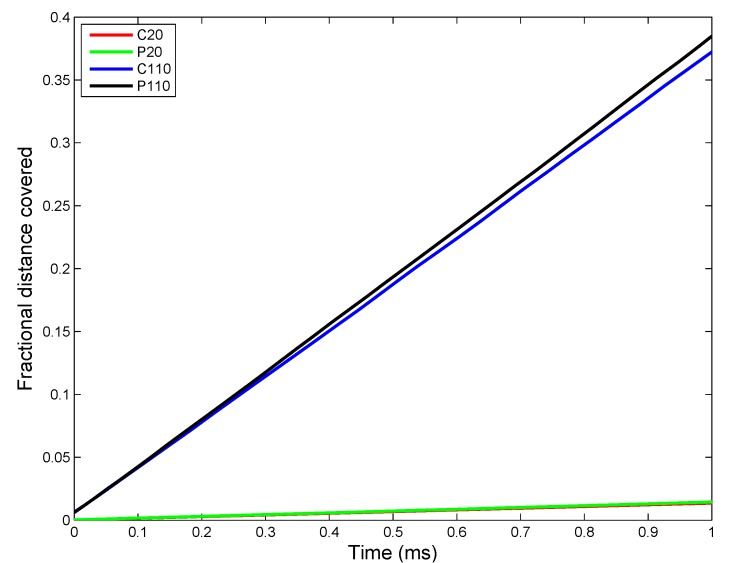
ADSRM simulation results of fractional distance covered by ENMs (ENM diameters of 20 and 110 nm; other ENM properties in Table 5) across the alveolar lining thickness (a value of one represents the bottom of the lining layer at the cellular surface, and a value of zero represents the top of the surfactant layer).

**Figure 9:**
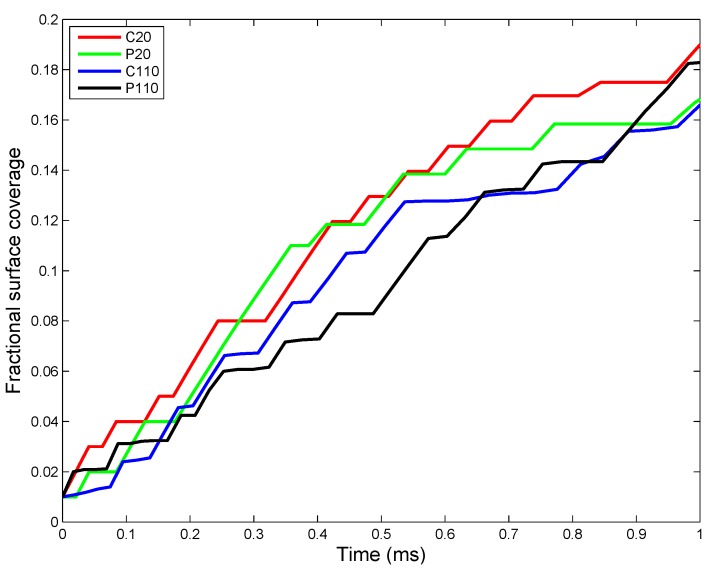
ADSRM simulation results of mean fractional surface coverage of ENMs (ENM diameters of 20 and 110 nm; other ENM properties in Table 5) by adsorbed surfactant phospholipids for four different types of silver ENMs.

**Figure 10:**
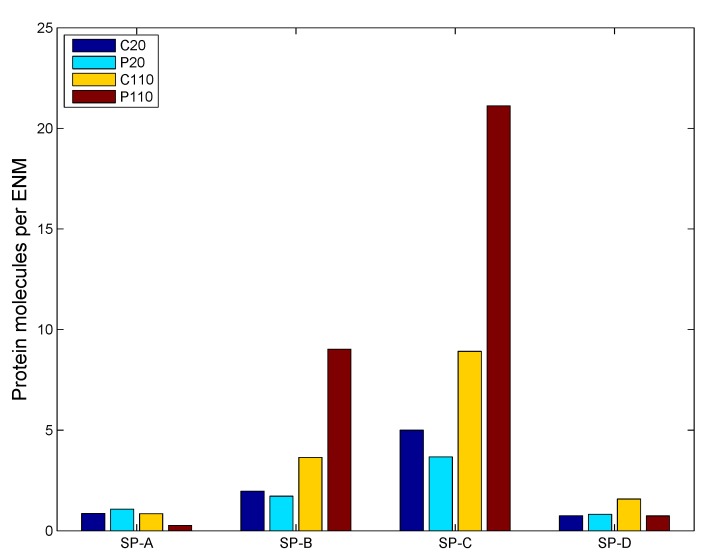
ADSRM simulation results of surfactant protein molecules adsorbed on different ENMs (ENM diameters of 20 and 110 nm; other ENM properties in Table 5).

Implementation of the ADSR Model for an actual *in vivo* system allows one to test the interplay of various parameters that have been estimated from *in vitro* studies. The results allow the estimation of average kinetic rate constants, which can then be used for higher level (tissue and organism level) models. Figure 7 shows the change in ENM diameter for the different types of ENMs considered, due to agglomeration. Larger ENMs (C110 and P110) seem to have a higher rate of agglomeration than the smaller ENMs. This might be counter-intuitive, since most studies [55] have demonstrated smaller particles to have a higher tendency to agglomerate than larger particles. However, such tissue-level studies compare particles based on identical mass doses of different particles. Equal mass doses of larger and smaller particles would result in significantly larger numbers of the smaller particles, resulting in a far higher number concentration, which would enhance the number of probable collisions between the particles per unit time. On a per particle basis, a larger ENM would have a higher van der Waals’ force of attraction than a smaller ENM, which explains the observed higher rates of agglomeration for C110 and P110. Among ENMs of the same size, PVP-coated ENMs have a slightly lower rate of agglomeration due to increased steric stabilization (quantified by the parameter $\eta_{\text{S}}$) by PVP molecules, as compared to citrate molecules. The results shown in Figure 7 are used to estimate an average rate of agglomeration as the increase in diameter per time unit. However, the model being stochastic, there are random variations in the results. The ADSRM model is run 10 times for each ENM, and various kinetic parameters are estimated for each run. The means of the estimated values are summarized in Table 6. Transport driven by gravitational forces tends to favor larger NPs as shown in Figure 8. However, due to ENM transport across the alveolar lining being ultimately driven by surface tension, there is no discernible difference in the transport rates among the different types of ENMs. Therefore, the average transport velocities for the different ENMs across the alveolar lining layer (Table 6) are identical. Figure 9 shows fractional surface coverage of the ENM surface due to surfactant phospholipid (PL) adsorption. Smaller ENMs seem to be coated with lipids faster, due to the fact that a single interaction between a vesicle and a smaller ENM might completely cover the smaller ENM, while for a larger ENM, this might require multiple interactions. Table 6 shows the rates for PL adsorption, which has been normalized by the surface area of the ENMs to cancel the effect of size. The PL adsorption rate has been quantified as the amount of PL adsorbed per unit surface area per time and is estimated based on the fractional surface coverage and considering a uniform PL bilayer of 4 nm on the ENMs. Comparison of the values shows that P20 has a slightly lower value (even lower than P110) of adsorption rate, which might be due to the higher number of coating molecules per unit surface area (Table S2 in Supplementary Materials) on P20 than on P110. Figure 10 shows the number of protein molecules adsorbed per ENM. SP-B and SP-C have a higher prevalence due to their close association with surfactant lipids. Furthermore, larger ENMs (C110 and P110) have a higher tendency to attract surfactant proteins due to larger available surface area.

**Table 6:** Estimated parameter values for ENM kinetics *in vivo*.

Parameter	C20	C110	P20	P110
Agglomeration rate (nm/s)	5.423 × 10${}^{-\text{6}}$	3.483 × 10${}^{-\text{5}}$	5.82 × 10${}^{-\text{6}}$	3.986 × 10${}^{-\text{5}}$
Transport rate (m/s)	7.481 × 10${}^{-\text{5}}$	7.481 × 10${}^{-\text{5}}$	7.481 × 10${}^{-\text{5}}$	7.481 × 10${}^{-\text{5}}$
Dissolution rate (per s)	4.25 × 10${}^{-\text{11}}$	6.375 × 10${}^{-\text{9}}$	5.525 × 10${}^{-\text{14}}$	9.52 × 10${}^{-\text{13}}$
Phospholipid (PL) adsorption rate (ng/nm${}^{\text{2}}$/s)	1.053 × 10${}^{-\text{7}}$	1.304 × 10${}^{-\text{7}}$	9.06 × 10${}^{-\text{8}}$	1.166 × 10${}^{-\text{7}}$
Surface active (SA) protein adsorption rate (nmol/nm${}^{\text{2}}$/s)	3.36 × 10${}^{-\text{18}}$	1.61 × 10${}^{-\text{19}}$	3.233 × 10${}^{-\text{18}}$	1.29 × 10${}^{-\text{19}}$
Collectin (C) adsorption rate (nmol/nm${}^{\text{2}}$/s)	2.66 × 10${}^{-\text{18}}$	1.55 × 10${}^{-\text{19}}$	3.737 × 10${}^{-\text{18}}$	8.472 × 10${}^{-\text{20}}$

#### 3.2.2. Sensitivity Analysis

**Figure 11:**
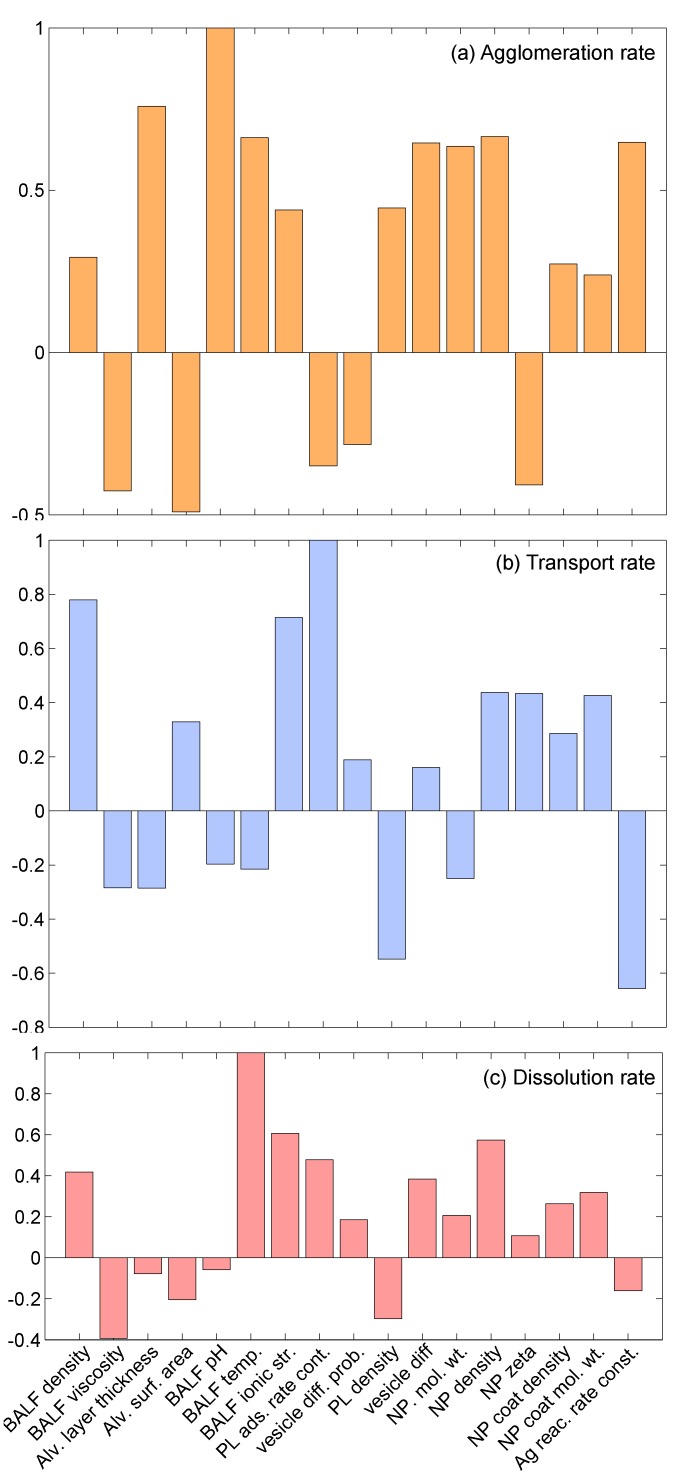
Sensitivity indices for 17 parameters corresponding to the *in vivo* implementation of ADSRM for three different outputs: (**a**) agglomeration rate; (**b**) transport rate; and (**c**) dissolution rate.

A local sensitivity analysis was conducted to understand the effect of various parameters on the final model results. The sensitivity analysis included a total of 17 parameters, which can be classified into three groups. The first group had seven parameters corresponding to the properties of the alveolar interface layer and included bronchoalveolar lavage fluid (BALF) density, BALF viscosity, BALF pH, BALF temperature, BALF ionic strength, alveolar layer thickness and alveolar surface area. The second group included four parameters corresponding to phospholipid (PL) adsorption: PL adsorption rate constant (α), PL vesicle diffusion probability (P${}_{\text{diff}}$), PL density and PL vesicle diffusivity. The third group consisted of parameters reflecting properties of the NPs and included: NP material molecular weight, NP material density, NP surface zeta potential, coating density, coating molecular weight and reaction rate constant. The sensitivity analysis was conducted by changing the value of one parameter at a time by 1%, while keeping values of all other parameters at their nominal values. The sensitivity index was calculated using the equation: $S_{\text{i}}=\left(V_{i}^{\text{*}}-V\right)/\left(V.\Delta p_{\text{i}}\right)$, where *V* represents the value of the particular variable corresponding to nominal values of all parameters, $V_{i}^{\text{*}}$ represents the value of the variable with the changed value of the parameter, $p_{\text{i}}$, and $\Delta p_{\text{i}}$ represents the fractional change in the value of $p_{\text{i}}$. Values of output variables might increase or decrease with the increase in values of parameters, and accordingly, the corresponding sensitivity indices might be positive or negative. Furthermore, due to the stochastic character of the model, values of the output variables change on every iteration. Accordingly, the model was run for 10 iterations, and the mean of the sensitivity indices was taken. Figure 11 shows the results of the sensitivity analysis for three output variables: agglomeration rate, transport rate and dissolution rate. The individual indices have been normalized by the highest valued index for a particular output variable. Overall, it can be said that the properties of the BALF strongly affect agglomeration rate of the NPs. For the transport rate, only BALF density and ionic strength seem to have a substantial effect. For the dissolution reaction rate, only BALF temperature and ionic strength have an effect. This should be expected, since the dissolution reaction is a chemical process and is affected by temperature and the ionic concentration of the medium. It can also be observed in Figure 11 that the stochastic parameters—PL adsorption rate constant and PL vesicle diffusion probability—have a relatively low effect on the output variables. The third group of parameters, consisting of physicochemical properties of NPs, is found to strongly affect agglomeration of NPs and only affect transport moderately.

## 4. Conclusions

The work described here presents an extension and implementation of the ADSR Model [7] for the pulmonary alveolar lining layer. An earlier version of the model had been developed [7] for *in vitro* cell cultures to assess ENM dosimetry to the cells. The problem of dosimetry is, however, more critical for *in vivo* toxicodynamic models. Generally, toxicokinetic and toxicodynamic models for NPs [15,17,57] approximate various physiological compartments via well-mixed compartments and mechanisms by linear, first-order kinetic rates. However, due to the size and unique properties of ENMs, their interactions with various biological molecules and cells often cannot be described by simple kinetic equations. This paper describes a stochastic DSMC algorithm to model the interactions of ENMs with each other and with biological agents in the alveolar lining layer, which forms a critical first line of defense against inhaled particulate matter. The model considers the agglomeration, transport and reaction of ENMs as treated in the earlier version of the model [7], and also includes surfactant lipid and protein adsorption on ENMs. Surfactant adsorption has been identified as a critical mechanism that regulates particle toxicodynamics and affects further uptake and clearance of the ENMs. Surfactant proteins have multiple roles, including promoting surface activity (SP-B, SP-C) and inducing immune response (SP-A, SP-D). All four protein types have been separately identified, and their adsorption on the ENMs is modeled here using the DSMC scheme. The model was first implemented for *in vitro* systems in order to parameterize the extended formulation utilizing published data. The model was subsequently applied to the human alveolar lining layer, considering an inhalation dose of four types of ENMs, to investigate the effects of size, as well as surface coating type.

*In vivo* toxicodynamics of NPs consists of multiple interactions between the particles themselves and also with cells and biochemical molecules. These interactions lead to agglomeration and reactions causing changes in the state of the particles. Changes in the shape, size and agglomeration state of NPs are known to affect the interaction of these NPs with various cells of biological systems [12,58]. The model described here considers the most important interactions and mechanisms relevant to particle toxicodynamics in the alveolar lining layer. The model utilizes mechanistic information available in the scientific literature and also relevant *in vitro* studies to simulate a phenomenon for which, at present, there are no *in vivo* measurements. However, the process of surface modification of NPs *in vivo* is very complex and involves a number of proteins, salts and other biomolecules [59]. The proteins corona on the NPs can also be distinguished into the hard corona and the soft corona, each of which have individual kinetics [21]. All of these interactions affect cellular uptake and trafficking in ways that are not yet fully elucidated. Future research into nano to bio interactions will probably produce novel information, which can be incorporated into multiscale frameworks, such as ADSRM, leading to improved modeling formulations. This will help us compare and contrast the effects of multiple processes and interactions simultaneously.

*In vivo* characterization of particokinetics in the pulmonary regions is extremely difficult due to the small size and time scales, which prevent an accurate and quantifiable description. The model described here will be utilized to inform and improve tissue-scale pulmonary toxicodynamic models involving ENMs. Based on a sensitivity analysis, it was found that the properties of the alveolar lining, the properties of the NPs, and those of the phospholipid vesicles of the alveolar lining are mostly responsible for the variation in NP kinetics in the alveolar layer. While the biological properties of the alveolar lining are fairly well characterized, NP properties exhibit wide variability. This calls for more accurate and reproducible estimation of NP properties, especially density, surface zeta potential and coating efficiency.

## Supplementary Materials

Supplementary materials can be accessed at: http://www.mdpi.com/2079-4991/5/3/1223/s1.
