# Supplementary material for: Modeling In Vivo Interactions of Engineered Nanoparticles in the Pulmonary Alveolar Lining Fluid
Source: Nanomaterials (Basel). 2015 Jul 22;5(3):1223–49. doi: 10.3390/nano5031223 (PMC4521411; doi:10.3390/nano5031223)
Supplement: Supplementary file 1 [file nanomaterials-05-01223-s001.pdf]

# Supplementary Information

## 1. Size Distribution of Lipid Vesicles

The size distribution of lipid vesicles is constructed based on available information regarding vesicular size in mammalian alveolar fluid. There are generally three broad types of vesicles based on size: small unilamellar vesicles (SUVs), large unilamellar vesicles (LUVs) and giant unilamellar vesicles (GUVs). The available information has been summarized in Table S1.

The upper and lower limits of the size ranges are taken as the 95% and 5% values of a log-normal distribution for vesicular size. Then, the subsequent mean and standard deviation for each type of vesicle are estimated. The measures for the respective log-normal distributions are combined with the respective percent compositions to construct an overall population of vesicles having SUVs, LUVs and GUVs with a size distribution representative of mammalian alveolar fluid. The figure shows the overall size distribution of lipid vesicles.

**Table S1.** Properties of surfactant lipid vesicles.

| Vesicle type | Percent composition * | Size range ** | Mean, SD (nm) |
|---|---|---|---|
| SUV | 43% | 20–50 nm | 35, 7.5 |
| LUV | 48% | 50–500 nm | 275, 112.5 |
| GUV | 9% | >1000 nm | 1000, 0 |

* From Goerke, 1998 [1]; ** From Notter, *Lung Surfactants* [2].

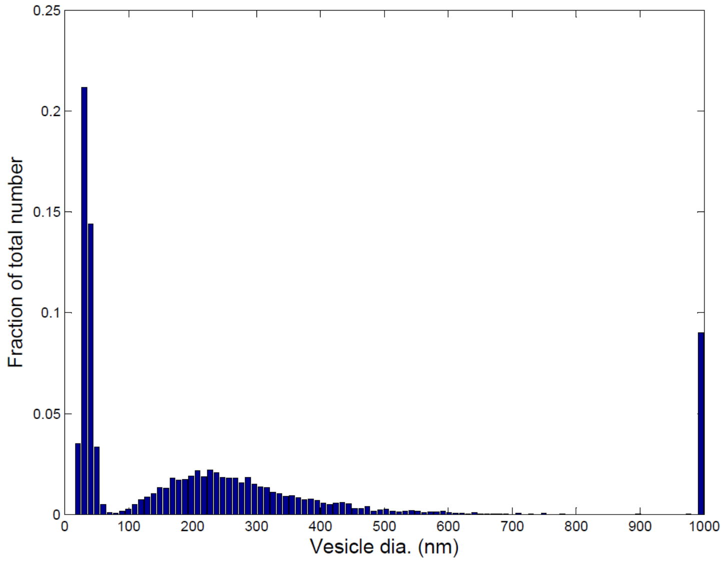


**Figure S1.** Size distribution of surfactant lipid vesicles.

## 2. Estimation of Steric Stabilization

Steric effects have been considered for coating molecules using the method shown by Damodaran [3], who used the following equation to estimate the repulsive energy due to steric effects:

$$E_{st} = \left(kTn_m L / s\right).\left[\left(2L / d\right)^{2.25} - \left(d / 2L\right)^{0.75}\right] \tag{S1}$$

where $k$ is Boltzmann's constant, $T$ is the absolute temperature, $n_m$ is the number of coating molecules per unit surface area of the ENM, $L$ is the chain length of the coating molecule, $s$ is the mean distance

between coating molecules and $d$ is the mean distance between interacting ENMs. Here, the mean distance between coating molecules, $s$, is given by $s = \sqrt{1/n_m}$ . The distance between interacting ENMs, $d$, varies as two ENMs approach each other, and hence, the total interaction energy is estimated by integrating over the entire distance, as was done by Mukherjee *et al.* [4] for attractive and repulsive electrical potentials.

Parameters $L$ and $n_m$ in Equation (S1) for citrate and PVP molecules are summarized in Table S2. The number of coating molecules per unit ENM surface area ($n_m$) is generally known to vary with ENM size. The values of $n_m$ for various sizes of ENMs have been estimated by Mukherjee *et al.* [4]. The molecular lengths of citrate and PVP molecules are taken from the literature (PVP from Zeng *et al.* [5] and citrate from Appelblat and Manzurola [6]).

**Table S2.** Coating molecule densities for different NPs.

| Parameters | C20 | C110 | P20 | P110 |
|---|---|---|---|---|
| $L$ | 6.73 A° * | 6.73 A° * | 16 nm ** | 64 nm ** |
| $n_m$ (molecules/m$^2$) | $8.94 \times 10^{18}$ | $4.92 \times 10^{19}$ | $4.36 \times 10^{19}$ | $2.9 \times 10^{19}$ |

* Based on an apparent molar volume of 96.24 cm$^3$/mol for trisodium citrate from Appelblat and Manzurola [6]; ** Based on 10 kDa and 40 kDa PVP chain length from Zeng *et al.* [5].

The steric repulsive potential estimated from Equation (S1) is normalized by the steric potential created by an uncoated Ag ENM. The molar volume of Ag is 10.335 cm$^3$ per mole. Considering an Avogadro number of molecules making up that volume and assuming an Ag atom as roughly spherical, the mean radius of the Ag atom, $r_{Ag}$, can be estimated as 1.6 A°. Empirical and calculated values of the atomic radius of Ag are also reported to be around 160 pm or 1.6 A° (Source: Webelements [7]). Number of Ag atoms present on the surface of an uncoated Ag ENM can be estimated as $n_m = 1/\pi r_{Ag}^2$ .

Therefore, using $L$ = 3.2 A° and $n_m = 1.24 \times 10^{19}$ atoms/m$^2$, we can get from Equation (S1) the value of $E_{Ag}$. The steric effect is quantified as $\lambda_{ST} = \frac{|E_{Ag}|}{|E_{coat}|}$.

The mutual interaction of ENMs consists of an attractive van der Waals' interaction potential and a repulsive interaction potential. Detailed expressions for these interaction potentials are provided in the original ADSRM article (Mukherjee *et al.* [4]). The repulsive interaction potential between the agglomerates can be expressed via the electric double layer (EDL) interaction potential equation that was developed using the linear superposition principle by Gregory [8] as:

$$\phi_R(h) = 128\varepsilon\pi\left(\frac{k_B T}{Ze}\right)^2\left(\frac{r_i r_j}{r_i + r_j}\right)\gamma^2 \exp(-\kappa h) \quad \text{(S2)}$$

Here, $\varepsilon$ is the permittivity of the medium, $Z$ is the valence of ions in the medium, $e$ is the elementary charge, $k_B$ is the Boltzmann's constant, $\kappa$ is the Debye–Hückel parameter and $\gamma$ is the reduced surface potential, which is a function of the surface zeta potential $\zeta$ of the particles. The effective repulsive potential, $\varphi_e$, is obtained by adjusting the electric repulsive potential $\varphi_R$ by the steric effect represented by $\lambda_{ST}$ as:

$$\phi_e(h) = \phi_R(h).\lambda_{ST}(h) \quad \text{(S3)}$$

## 3. Estimation of Zeta Potentials

The zeta potential of lipid-adsorbed ENMs has been estimated using a weighed function as:

$$\zeta = \zeta_L.\theta + \zeta_0(1-\theta) \quad \text{(S4)}$$

where $\zeta_0$ is the zeta potential of the ENM without lipid adsorption, $\zeta_L$ is the reduced zeta potential due to lipid adsorption and θ is the fractional surface coverage of the ENM by lipids. Allam *et al.* [9] measured zeta potentials of nanoparticles, with and without the presence of lipids.

**Table S3.** Surface zeta potential (in mV) for NPs.

| **ζ for uncoated NPs** | **−32.4** | **−40.7** | **−47.1** | **−11.2** | **−12.3** | **−23.3** | **−10.3** | **−10.8** | **−22.5** |
|---|---|---|---|---|---|---|---|---|---|
| ζ for lipid coated NPs | −11.9 | −15.6 | −19.1 | −4.5 | −5.5 | −7.4 | −2.2 | −3.4 | −5.2 |

Measured values of zeta potential for coated and uncoated NPs from Allam *et al.* [9]; From these measurements, the average value of $\zeta_L/\zeta_0$ was estimated as 0.34; Zeta potentials of various types of uncoated ENMs are summarized in the main article.

## 4. Protein Adsorption and Desorption

Zhdanov and Kasemo [10] modeled protein adsorption and desorption on lipid bilayers and how the adsorption and desorption rates ($k_a$ and $k_d$) varied with lipid surface coverage. The measurements for adsorption and desorption were used to fit a function for $k_a$ and $k_d$ as follows:

$$k_a = k_a^0.(1+\beta_a \theta^{n_a}) \quad \text{(S5)}$$

$$k_d = k_d^0.(1+\beta_d \theta^{n_d}) \quad \text{(S6)}$$

Here, $k_a^0$ and $k_d^0$ are the values of $k_a$ and $k_d$ obtained by extrapolation of the data shown above for θ = 0. Without the presence of lipids (θ = 0), there would be no preference for any of the proteins regarding adsorption, and hence, the values of $k_a^0$ and $k_d^0$ would be the same. The exact values do not matter here, as they are normalized in the model and used as relative probabilities. The values of the fitted parameters for the four surfactant proteins are shown below:

**Table S4.** Adsorption and desorption parameters for surfactant proteins.

| **Protein** | ***L*** | $k_a^0$ | $k_d^0$ | $\beta_a$ | $\beta_d$ | $k_a$ | $k_d$ |
|---|---|---|---|---|---|---|---|
| SP-A | 2 | $3.16 \times 10^{-4}$ | $2 \times 10^7$ | $1.5 \times 10^4$ | $2.5 \times 10^{-7}$ | 1.79 | −3.2 |
| SP-B | 4 | $3.16 \times 10^{-4}$ | $2 \times 10^7$ | $4.58 \times 10^7$ | $2.3 \times 10^{-3}$ | 3.92 | −1.09 |
| SP-C | 4 | $3.16 \times 10^{-4}$ | $2 \times 10^7$ | $4.58 \times 10^7$ | $2.3 \times 10^{-3}$ | 3.92 | −1.09 |
| SP-D | 2 | $3.16 \times 10^{-4}$ | $2 \times 10^7$ | $1.5 \times 10^4$ | $2.5 \times 10^{-7}$ | 1.79 | −3.2 |

## 5. Calculation of Protein Diffusion Coefficients

The estimation of diffusion coefficients of proteins involves multiple parameters, such as the molecular weight *M*, partial specific volume $\bar{V}$ and the sedimentation coefficient, *s*, which are related by the Svedberg equation [11] as shown below:

$$D = \frac{RTs}{M(1 - \bar{V}\rho)} \tag{S7}$$

The sedimentation parameter, *s*, is given by: $s = v/a$, where $v/a$ is the ratio of the volume-to-area of the protein molecule concerned. Young *et al.* [11] used the Svedberg equation to derive an empirical correlation for proteins, which can be written as:

$$D = (7.51 \times 10^{-8}) \cdot T\bar{V}^{-1/3} / \eta \tag{S8}$$

where $\bar{V}$ is the partial specific volume of the protein under consideration, $\eta$ is the viscosity of the medium and $T$ is the absolute temperature.

## References

1. Goerke, J. Pulmonary surfactant: Functions and molecular composition. *Biochim. Biophys. Acta* **1998**, *1408*, 79–89.
2. Notter, R.H. *Lung Surfactants: Basic Science and Clinical Applications*; Marcel Dekker Inc: New York, NY, USA, 2000; Volume 149.
3. Damodaran, S. *Food Proteins and Their Applications*; Marcel Dekker Inc: New York, NY, USA, 1997.
4. Mukherjee, D.; Leo, B.; Royce, S.; Porter, A.; Ryan, M.; Schwander, S.; Chung, K.; Tetley, T.; Zhang, J.; Georgopoulos, P. Modeling physicochemical interactions affecting *in vitro* cellular dosimetry of engineered nanomaterials: Application to nanosilver. *J. Nanopart. Res.* **2014**, *16*, 1–16.
5. Zeng, X.; Zhou, B.; Gao, Y.; Wang, C.; Li, S.; Yeung, C.Y.; Wen, W. Structural dependence of silver nanowires on polyvinyl pyrrolidone (PVP) chain length. *Nanotechnology* **2014**, *25*, 495601.
6. Apelblat, A.; Manzurola, E. Apparent molar volumes of organic acids and salts in water at 298.15K. *Fluid Phase Equilibria* **1990**, *60*, 157–171.
7. Webelements. Available online: http://www.webelements.com (accessed on November 2014).
8. Gregory, J. Approximate expressions for retarded van der Waals interaction. *J. Colloid Interface Sci.* **1981**, *83*, 138–145.
9. Allam, A.A.; Sadat, M.E.; Potter, S.J.; Mast, D.B.; Mohamed, D.F.; Habib, F.S.; Pauletti, G.M. Stability and magnetically induced heating behavior of lipid-coated $Fe_3O_4$ nanoparticles. *Nanoscale Res. Lett.* **2013**, *8*, 426.
10. Zhdanov, V.P.; Kasemo, B. Protein adsorption and desorption on lipid bilayers. *Biophys. Chem.* **2010**, *146*, 60–64.
11. Young, M.E.; Carroad, P.A.; Bell, R.L. Estimation of diffusion coefficients of proteins. *Biotechnol. Bioeng.* **1980**, *22*, 947–955.

© 2015 by the authors; licensee MDPI, Basel, Switzerland. This article is an open access article distributed under the terms and conditions of the Creative Commons Attribution license (http://creativecommons.org/licenses/by/4.0/).
